# Microstructure, Mechanical Properties and Tribological Properties of NiAlComposites Reinforced by CrMnFeCoNiHigh-Entropy Alloy

**DOI:** 10.3390/ma11101850

**Published:** 2018-09-28

**Authors:** ShuQiang Zhou, XinYu Liu, Yi Xu

**Affiliations:** 1 School of materials Science & Engineering, Southwest Jiaotong University, Chengdu 610031, China; zhusuqiang@163.com; 2 Chengdu Advanced Metal Materials Industry Technology Research Institute Co, Ltd., Chengdu 610031, China; cgvermouth@sina.cn

**Keywords:** NiAl-HEA composites, microstructure, mechanical properties, tribological properties

## Abstract

NiAl-based composites reinforced by CrMnFeCoNi high-entropy alloy (HEA) particles were fabricated by mechanical alloying (MA) and spark plasma sintering (SPS). The microstructure, mechanical, and tribological properties of the NiAl-HEA composites were investigated. Microstructural analyses show that after SPS, the HEA phase homogenously distributed in the NiAl matrix. Non-uniform diffusion of various elements occurred during the high temperature sintering process. Transmission electron microscope (TEM) observation of the composites revealed that many nano particle of Al_2_O_3_ generated at the grain boundary. The yield strength significantly increased after adding HEA particles. The compressive strength of the composites increased with the contents of HEA increasing, which should be attributed to the second phase hardening effect of HEA particles and fine grain strengthening effect. The composite of 10 wt.% HEA exhibited significant room temperature compressive properties, with the ultimate compressive strength of 2692 MPa and the compressive strain of 34.2%, respectively. The results of the wear tests show that the addition of HEA will reduce the wear resistance of composites to some extent and slightly increase the coefficients of friction (COFs) of the composites.

## 1. Introduction

The NiAl intermetallic has been recognized as a candidate of high temperature structural materials, due to its excellent physical and mechanical properties, such as low density (5.9 g/cm^3^), high melting points (1911 K), and excellent oxidation resistance up to 1573 K, as well as good thermal conductivity (2–4 times higher than conventional nickel-based alloys) [1,2,3,4]. However, two major weaknesses limited its applications due to the poor ductility at ambient temperature and low strength at high temperature [5]. Preparing materials into composites is one of the effective ways to improve the properties of materials. A great many of efforts, such as ceramic reinforcement [6,7] and oxide-dispersion strengthening (ODS) [5,8,9] have been offered to overcome these limitations. Traditionally using ceramic reinforcement particles displayed some disadvantages that cannot be ignored, such as poor wetting with the matrix, which caused for porosity that was localized at the matrix-reinforcement interfaces [10].

High entropy alloys (HEAs), which are a new class of materials, are known for their good structural stability, high ductility, and exceptional high-temperature strength. It usually contains four or five metallic elements with nearly equiatomic ratios. They are also recognized as the promising candidate for structural application [11].

Recently, HEAs have been used to reinforce metallic matrixes due to their exceptional properties [12,13]. For example, FeCoCrAlCuV*_x_*Ni HEA has been proven to create good bonding with the substrate and it can effectively improve the mechanical properties of the matrix [14]. Fu Z et al. [15] also reported that the addition of TiNiFeCrCoAl HEA into TiB_2_ can significantly enhance the densification of the composite, thus improving the mechanical performance.

CrMnFeCoNi, as one of the successful HEAs, has attracted many attentions, especially its good plasticity and high temperature stability [11]. In this study, NiAl composites reinforced with CrMnFeCoNi HEA have been designed and then produced by powder metallurgy (PM). For many potential applications of NiAl alloy, sliding friction is inevitable, so it is necessary to study the friction properties of NiAl composites. The microstructure, mechanical properties and room temperature (RT) tribological properties of the composites were investigated.

## 2. Materials and Methods

**Figure 1:**
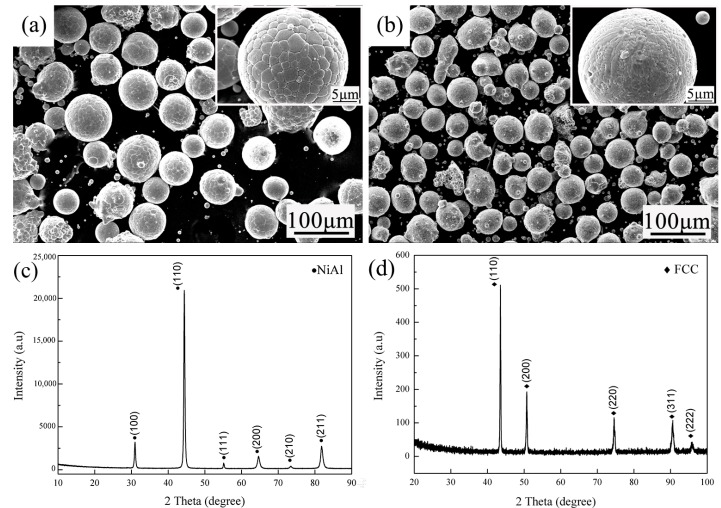
Scanning electron microscopy (SEM) images of the gas atomized NiAl powders (**a**) and CrMnFeCoNi high-entropy alloy (HEA) powders (**b**); X-ray diffraction (XRD) patterns of NiAl (**c**); and CrMnFeCoNi HEA (**d**).

For the production of the NiAl-HEA composites, the following powders were used: spherical gas-atomization NiAl alloy powders (Figure 1a) and CrMnFeCoNi HEA powders with particle size distribution that is in the range of 5–60 μm (Figure 1b). The corresponding X-ray diffratometer (XRD) patterns of NiAl alloy powders and CrMnFeCoNi HEA powders are shown in Figure 1c,d.

NiAl powders were blended with 0 wt.%, 5 wt.%, 10 wt.% and 15 wt.% of CrMnFeCoNi HEA powders in a planetary ball mill (QM-3SP4, Nanjing University Instrument, Nanjing, China) with a container and balls (10 mm diameter) made of stainless steel. For the convenience of description, the composites of the four components are called by 0 wt.% HEA, 5 wt.% HEA, 10 wt.% HEA, and 15 wt.% HEA, respectively.

The mechanical mixing process was conducted under the following conditions: rotational speed 250 rpm, ball to powder ratio (BPR) 10:1, and mixing time 5 h in argon atmosphere. At last, the as-milled powders were sintered while using spark plasma sintering (SPS-1050, Japan Fuji Electric Machinery Co., Ltd., Aichi Prefecture, Japan) at 1200 °C for 5 min under 50 MPa uniaxial pressure with a vacuum of 1 × 10^−3^ Pa.

The density of the sintered samples was measured by Archimedes method. The microhardness was measured by a Vickers hardness tester (HVS-1000B, Russell Fraser Sales Pty Ltd., Kirrawee, Australia) while using a load of 100 gf and a dwell time of 15 s. The powders and sintered samples were characterized via X-ray diffraction (XRD, PANalytical, Almelo, The Netherlands) with a Cu-Kα (λ = 0.154 nm) radiation for phase identification. The step size and the 2θ set as 0.013° and 10–90°, respectively. The software that was used to phase identification is MDI Jade 6 (MDI, Burbank, CA, USA). The microstructure of the compacted samples was examined by means of optical microscopy (OM, Olympus corporation Olympus, USA) and scanning electron microscopy (SEM, FEI, Hillsboro, OR, USA) with energy dispersive spectroscopy (EDS, Oxford instruments, Oxford, UK) detector. The specimens were polished and chemically etched with an acidic mixture (HNO_3_:HCl = 1:4) [16]. The slices for transmission electron microscope (TEM, JEOL, Akishima, Japan) were polished to 50 μm and then were shaped into 3 mm in diameter, followed by ion milling to perforation.

The compressive specimens with size of Φ 3.5 mm × 7 mm were cut from compacted composites. The compression tests were conducted in a compressive testing machine (UTM5105, Shenzhensuns, Shenzhen, China) at room temperature (RT), with an initial strain rate of 1 × 10^−3^ s^−1^. Each test was repeated for three times. The sliding wear tests were performed by friction and wear machine (CETRUMT-2, Bruker, Billerica, MA, USA). The test loads were 5 N, 10 N, and 15 N, respectively. The sliding speed was 5 mm/s and the test time set as 1500 s. The wear losses were calculated by NanoMap-Dual 2D profilometer (aep Technology, Santa Clare, CA, USA). The analysis of worn surfaces was performed by SEM.

## 3. Results and Discussion

### 3.1. Microstucture

**Figure 2:**
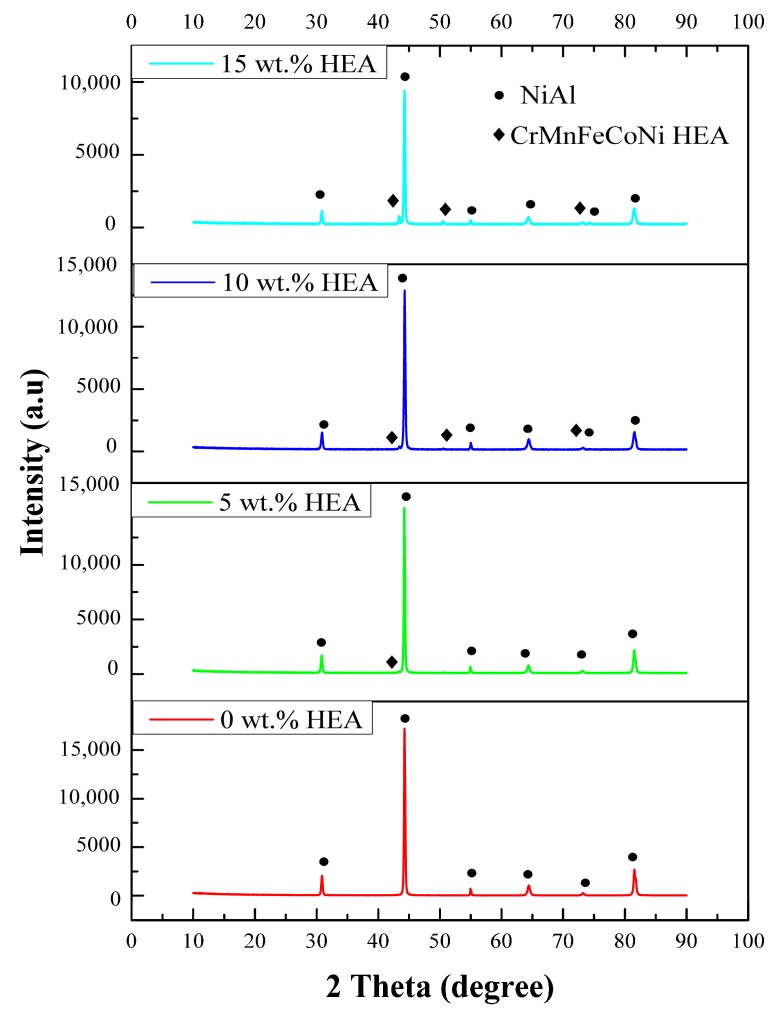
XRD patterns of the composites after sintering.

The XRD patterns of sintered NiAl-HEA composites with different weight fractions of the reinforcement are shown in Figure 2. The intensity of the NiAl diffraction peaks decreases with the increase of the HEA’s amounts, because the relative content of NiAl decreases. The NiAl phase and FCC HEA phase reveal a lattice parameter of 2.883 Å and 3.519 Å, respectively. Moreover, the diffraction peaks of NiAl ((100), (110), (111), (200), (210), (211)), and CrMnFeCoNi HEA ((110), (200), (220)) can be observed, and no diffraction peaks belonging to additional phases can be detected. These changes indicated that there was no reaction between the NiAl matrix and reinforcement, or the amounts of new phase was too low to be detected by XRD. This should be ascribed to the rapid sintering rate and short sintering periods.

**Figure 3:**
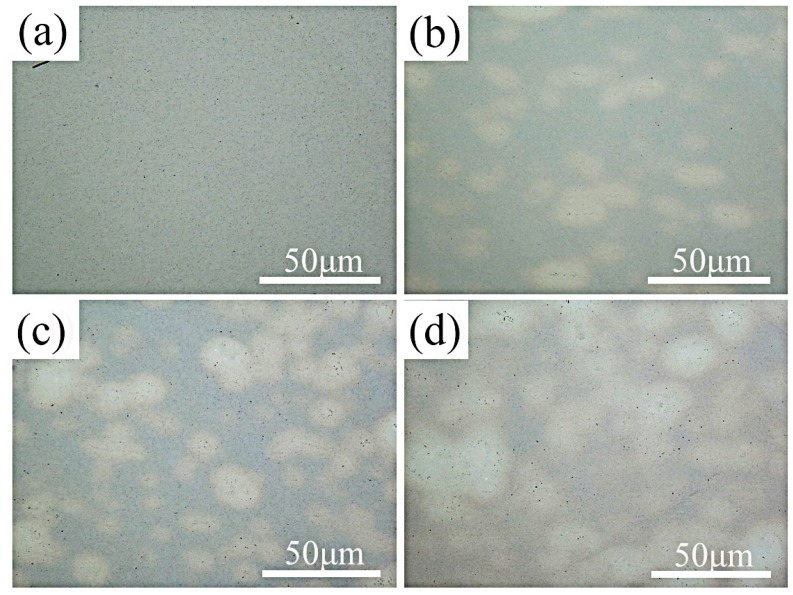
OM images of the composites: (**a**) 0 wt.% HEA; (**b**) 5 wt.% HEA; (**c**) 10 wt.% HEA; and (**d**) 15 wt.% HEA.

Figure 3 shows the optical microscope (OM) images of the composites’ cross-section with different weight fractions of the HEA reinforcement. The optical micrographs display a microstructure consisting of bright phase and gray phase, showing that the HEA distributed homogeneously in the NiAl matrix. There is no clear interface between bright phase and gray NiAl matrix. Different from the ceramic reinforcement [10], no obvious porosity was observed between the matrix and the enhancement phase.

**Figure 4:**
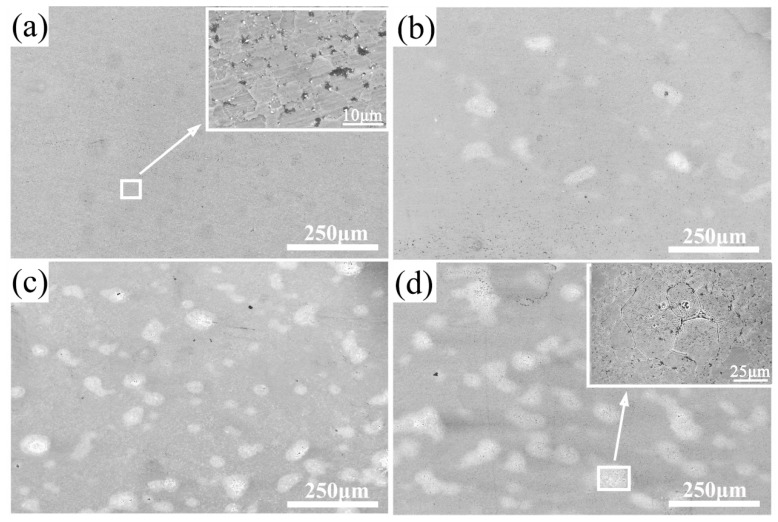
SEM images of NiAl-HEA composites after etched: (**a**) 0 wt.% HEA; (**b**) 5 wt.% HEA; (**c**) 10 wt.% HEA; and (**d**) 15 wt.% HEA.

The SEM images of NiAl-HEA composites after being etched are shown in Figure 4. The grain size of the matrix is ranging from 2 μm to 5 μm (inset image from Figure 4a), but the grains of bright phase are much larger than the matrix, reaching 20–40 μm (inset image from Figure 4d). The reason for this phenomenon may be contributed to the difference ductility of these two materials [17,18]. NiAl is a brittle intermetallic compound, while CrMnFeCoNi HEA has good ductility. During the ball milling process, the NiAl alloy powders were directly broken into smaller particles, while for the HEA powders, plastic deformation occurred.

**Figure 5:**
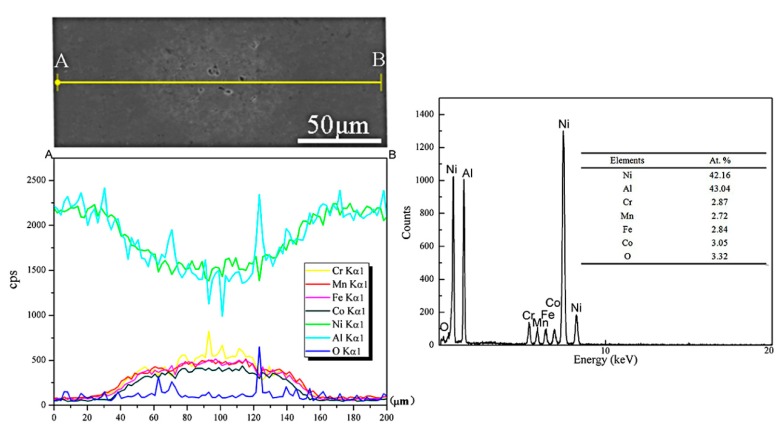
Energy dispersive spectroscopy (EDS) line analysis along line AB (10 wt.% HEA).

In order to explore the diffusion of different elements among the CrMnFeCoNi HEA phase and NiAl matrix, EDS elemental lines analyses were used, as shown in Figure 5. EDS line analyses showed that the dark gray areas were enriched in Ni and Al, which suggested that the dark gray areas were NiAl matrix. While the light gray areas are enriched in Cr, Mn, Fe, and Co, so it can be confirmed that the light gray areas are HEA phase. From Figure 4, it also can be observed that the concentration of Cr was different from Mn, Fe, Ni, and Co, which indicates that the elemental segregation of Cr may occur during the high temperature sintering process. According to the Refs [19,20], the researchers’ experiment results showed that if the elements are listed in the order of decreasing diffusion, the sequence is Mn, Cr, Fe, Co, and Ni. The diffusion rate of Cr is higher than Fe, Co and Ni in CrMnFeCoNi HEA, which may cause the different concentration of Cr. Different from Ref [21], the concentration of each element has a slowly changing trend rather than a sharply change. The content of elements did not change dramatically with distance, which indicated that significant elemental diffusion has occurred between the matrix and the HEA phase. The concentration changes of O and Al suggested that there may be produced Al_2_O_3_ inside the HEA phase.

**Figure 6:**
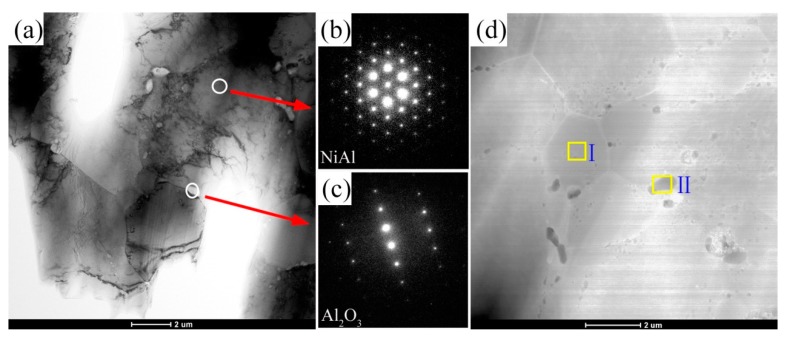
Transmission electron microscope (TEM) images of NiAl alloy (0 wt.%HEA): (**a**) microstructure of NiAl, (**b**,**c**) selected area diffraction (SAD) patterns of NiAl and Al_2_O_3_ from (**a**) and STEM image (**d**).

**Figure 7:**
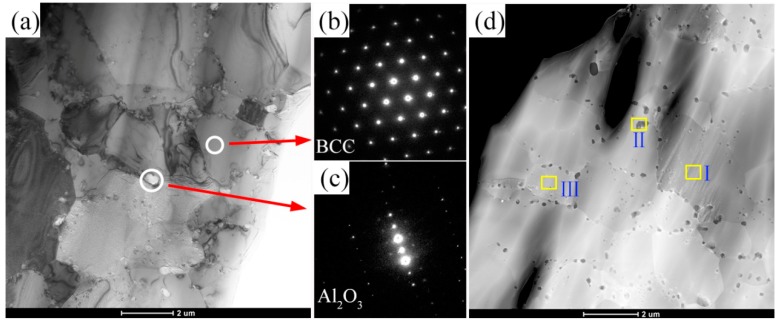
TEM images of NiAl composite reinforced with 10 wt.% HEA: (**a**) microstructure of 10 wt.% HEA, (**b**,**c**) SAD patterns of solid solution with the BCC structure and Al_2_O_3_ from (**a**), (**d**) STEM image.

For a further understanding the effect of addition CrMnFeCoNi HEA on the microstructure of the NiAl-HEA composites, TEM was used, as shown in Figure 6 and Figure 7. From Figure 6a, a small amount of nano-sized particles were generated in the NiAl grain boundary (0 wt.% HEA). The selected area diffraction (SAD) pattern showed the nano-sized particle phase is Al_2_O_3_ (Figure 6c). The SAD pattern from the solid ring in Figure 7a shows the solid solution with the BCC structure. In addition, the grain size of 10 wt.% HEA composite is smaller than 0 wt.% HEA (Figures 6d and Figures 7d), because Al_2_O_3_ particles might restrict the grain growth during the sintering process [22,23].

The presence of a small amount of Al_2_O_3_ in the NiAl alloy that was prepared by the method of PM is a common phenomenon, due to the reaction between the NiAl and oxygen during the sintering process [2,10,17]. As can be seen from the comparison between 0 wt.% HEA and 10 wt.% HEA composites, the addition of HEA will produce more Al_2_O_3_ particles. Because the atomic radius of Cr (0.1249 nm), Mn (0.1366 nm), Fe (0.1241 nm), and Co (0.1253 nm) are close to Ni (0.1246 nm) and Al (0.1432 nm). Transition metal elements, such as Fe and Ti, alloying decreases the solubility of Al and increases the solubility of Ni in NiAl [24,25]. Under the high temperature sintering condition, the reaction diffusion occurred between the two phases’ interfaces. The atoms of HEA diffused to the NiAl lattice and displaced part of the Al atoms at the phase interface. The similar results were also reported by Anderson, I. M. et al. [26]. Their experiment shows that most Fe atoms occupy the Ni sublattice, and some Fe atoms occupy the Al sublattice, necessitating the production of vacancies on the Ni sublattice to maintain the site ratio. Due to the reaction diffusion, the Al atoms combine with the O atoms to form Al_2_O_3_, which can be proved by EDS line analysis (Figure 5).

The chemical composition from areas I and II in Figure 6d, as measured by EDS, is shown in Table 1. EDS analysis of small particles, location II (Figure 6d), suggested that the particle should be the Al_2_O_3_, which is consistent with the results of SAD, Figure 6c. Additionally, the chemical composition from areas I and III showed a small amount of HEA elements are presented in the matrix grains (Figure 7d), indicating the diffusion of the elements between matrix and reinforcement. Also, chemical diffusion between matrix and reinforcement phase is often reported by other researchers for different types of composites [27,28].

### 3.2. Mechanical Properties

Table 2 presents the density and microhardness of sintered composites. The density of composites increases with increasing HEA contents, because the density of HEA is higher than that of NiAl. The 15 wt.% HEA composite has the highest density of 6.12 g/cm^3^. The microhardness of composites has the tendency of increasing with HEA’s contents increasing. 15 wt.% HEA composite has the highest hardness value (525 HV). According to Hall-Petch relation and previous TEM images (Figures 6d and Figures 7d), the finer grain size will lead to the higher hardness.

**Figure 8:**
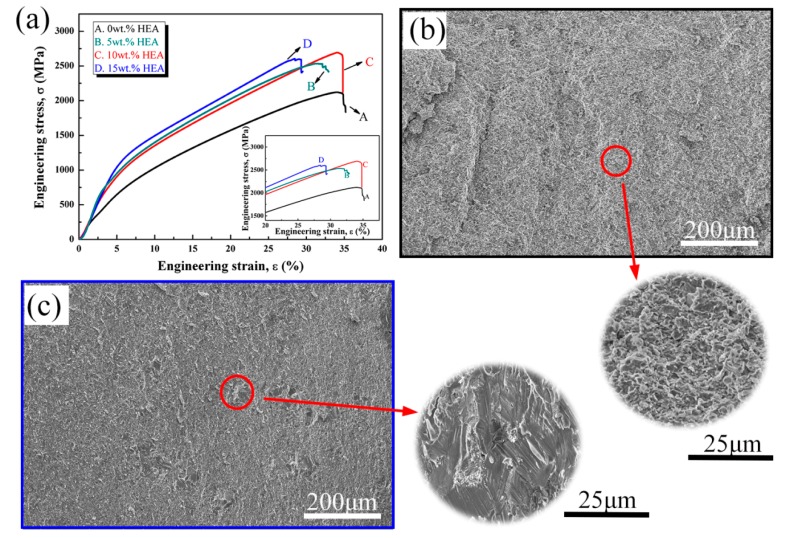
Compression stress-strain curves (**a**) and the fracture surface images: (**b**) 0 wt.% HEA, and (**c**) 15 wt.% HEA.

The room temperature compression behaviour of the composites is presented in Figure 8a. Table 2 also summarizes the yield strength (YS) and ultimate compressive strength (UTS) of the composites. The YS and UTS increase with amounts of HEA in the NiAl matrix. This indicates that the addition of HEA can result in an enhancement of the composites. The YS and UTS of composites increase from 779 MPa to 1566 MPa and from 2121 MPa to 2692 MPa, respectively, with increasing weight fractions of the reinforcement. However, no significant change in the compressive strain is observed by adding the HEA reinforcement particles. The composite of 10 wt.% HEA has the highest compression strain, reaching 34.2%.

Second phase hardening effect of HEA results in the difficulty of dislocations movement. Particle clustering produces high local stress concentration, thus it provides more crack nucleation sites and lower energy propagation routes. All compressive strains of composites are equal to or lower than that of 0 wt.% HEA. The similar results were also reported by Refs [13,29]. Brittle interfacial reaction products are commonly generated in metal matrix composites [30]. According to the STEM images, as shown in Figure 6 and Figure 7, many nano particles located in the grain boundary. When compared with 0 wt.% HEA, the numbers of nano particles at the grain boundary is significantly increased after the addition of HEA and the grain size is decreased, which lead to the increase of yield strength. Figure 8 shows the fracture surfaces of 0 wt.% HEA and 15 wt.% HEA composites, respectively. The fracture mode of 0 wt.% HEA is intergranular fracture (Figure 8b). The HEA phase of 15 wt.% HEA composite showed a river-like pattern, which indicates the plasticity of HEA phase decreases after high temperature reaction diffusion.

### 3.3. Tribological Properties

**Figure 9:**
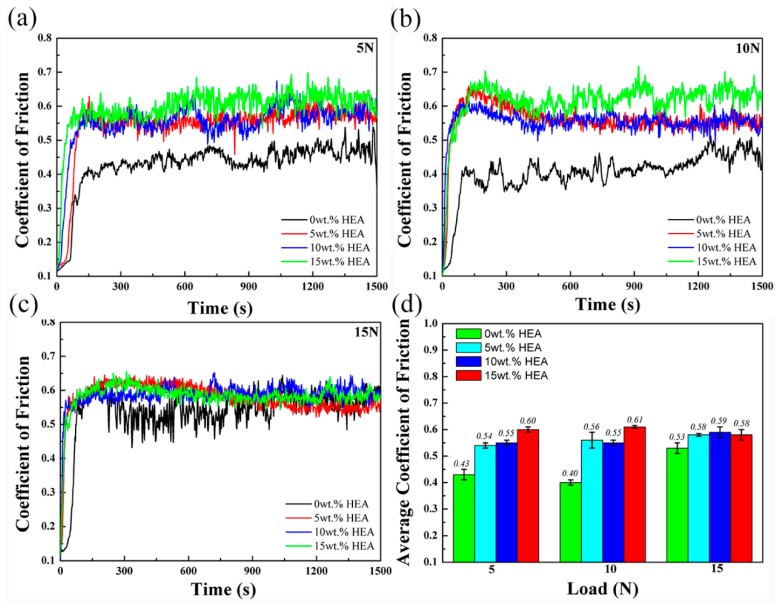
Coefficients of friction (COFs) under different loads: (**a**) 5N; (**b**) 10N; (**c**) 15N; and (**d**) average COFs.

The coefficients of friction (COFs) were obtained under different loads, as shown in Figure 9. Under the load of 5N, the average COF of 0 wt.% HEA was 0.43, while the average COF of 15 wt.% HEA increased to 0.60. The rest of two loads showed the same tendency. When the load increased to 15N, the COFs of four composites tend to be equal.

**Figure 10:**
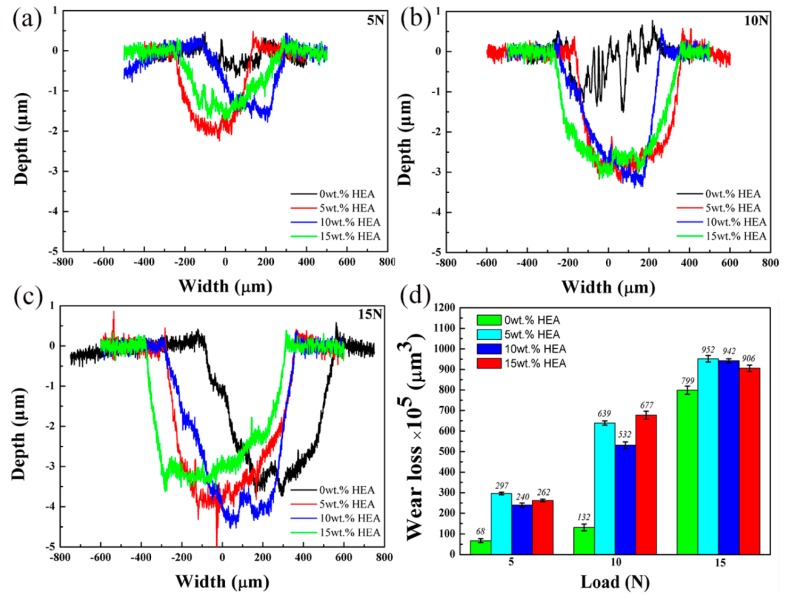
Cross-section profiles and wear losses of the worn plates: (**a**) 5N cross section profiles; (**b**) 10N cross section profiles; (**c**) 15N cross section profiles; and (**d**) wear losses.

Figure 10 shows the wear losses of these composites. The wear losses were estimated using the following equation [31]:

*WL* = *S_cs_× D* (1)

where *WL* is the wear loss, *S_cs_* and *D* mean the average of the wear scar cross-sectional area and wear distance, respectively. As Figure 10d shown, the wear losses of 0 wt.% HEA are the lowest in the all loads; when the load is small (5N and 10N), the addition of HEA will greatly reduce the wear resistance of the composites.

**Figure 11:**
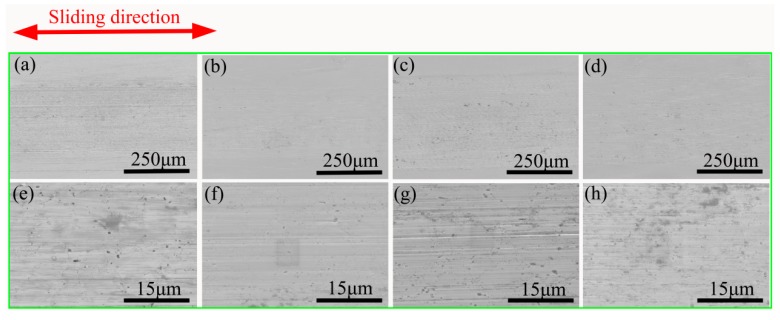
The wear surfaces of four composites tested under the load of 5 N: (**a**,**e**) 0 wt.% HEA; (**b**,**f**) 5 wt.% HEA; (**c**,**g**) 10 wt.% HEA; and, (**d**,**h**) 15 wt.% HEA, respectively.

**Figure 12:**
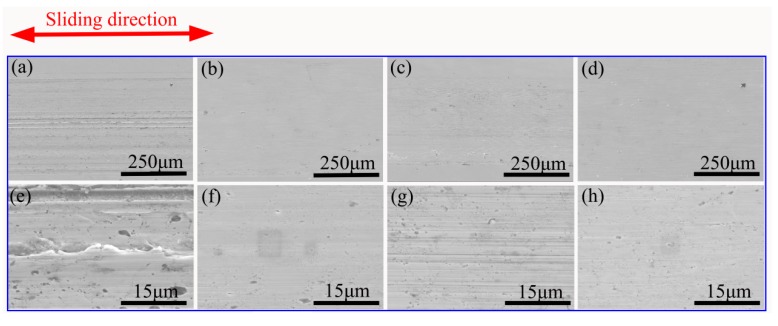
The wear surfaces of four composites tested under the load of 15 N: (**a**,**e**) 0 wt.% HEA; (**b**,**f**) 5 wt.% HEA; (**c**,**g**) 10 wt.% HEA; and, (**d**,**h**) 15 wt.% HEA, respectively.

In order to invesitigate the wear mechanism, the worn surfaces of the composites were analyzed by SEM. Figure 11 and Figure 12 show SEM micrographs of the wear surfaces after wear test under the loads of 5 N and 15 N. As shown in Figure 11, the wear surface of composites tested at 5 N was no significant difference, and a small amount of furrows can be seen, which indicates that the wear mechanism of the four composites under 5N load is mainly abrasive wear. When the load increased to 15 N, there were abundant parallel grooves and wear particles on the surface of the 0 wt.% HEA (Figure 12a,e). The wear mechanism is abrasive wear.

The surface of the NiAl alloy has a dense oxide film, which has a certain lubricating effect on the friction surface [31,32]. The addition of HEA reduces the area of the oxide film on the surface of the composites, and the lubrication of the oxide film decreases, resulting in the increase of the COFs. However, when the load increased to 15 N, the time for destroying the surface oxide film is shortened, so the lubrication effect is lowered, and the COFs of the four composites tend to be the same.

**Figure 13:**
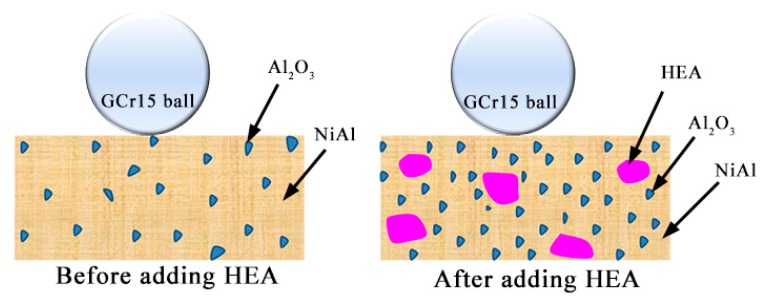
Schematic diagram of Al_2_O_3_ particle content of composites before and after the HEA addition.

The mechanism for friction and wear of the composites can be assisted by Figure 13. According to the Ref [33], the local particle contiguity and the possible formation of a continuous particle network confined the deformation of the composites. The interparticle distance may exert an influence on the trobological properties. Previous STEM images showed the interpaticle distance of the 10 wt.% HEA was shorter than that of the matrix (0 wt.% HEA), which will increase the COFs of the composites to some extent. Meanwhile, the hard oxide particles will result in promoting the excessive wear of softer matrix phase.

## 4. Conclusions

The CrMnFeCoNi high entropy alloy particles reinforced NiAl composites were prepared by MA and SPS process. The microstructure, mechanical, and tribological properties of the composites were investigated. Based on the test and analysis, the following conclusions can be drawn:

1. The distribution of HEA phase is relatively uniform after SPS. Under the high temperature SPS process, elemental diffusion occurs between the matrix phase and the HEA reinforcement phase. The addition of HEA will decrease the grain size of the composites and generate some Al_2_O_3_ particles at the grain boundary.

2. The density and hardness of the composites increase with the increasing of HEA content. Both the ultimate compressive strength and yield strength increase remarkably with an increasing weight fraction of the HEA. The YS and UTS of composites increase from 779 MPa to 1566 MPa and from 2121 MPa to 2692 MPa, respectively, which should be attributed to the second phase hardening effect and fine grain strengthening effect.

3. The addition of HEA will slightly increase the COFs of composites and accelerate the abrasive wear, and the reason can be attributed to the reduction of oxide film on the surface of NiAl matrix and the decrease of the interpaticle distance of hard abrasive Al_2_O_3_ particles.

## Figures and Tables

**Table 1:** Chemical composition of different areas in the sample of 0 wt.% HEA and 10 wt.% HEA.

Sample	Place of Analysis	Element (at.%)						
		Ni	Al	Cr	Mn	Fe	Co	O
0 wt.% HEA	Figure 5dI	49.21	51.79	-	-	-	-	-
	II	-	33.55	-	-	-	-	66.45
10 wt.% HEA	Figure 6dI	27.77	41.51	1.21	0.95	1.05	0.38	27.10
	II	-	36.07	-	-	-	-	63.92
	III	53.01	30.28	4.22	2.72	3.33	3.72	2.68

**Table 2:** Density, hardness, and compressive properties of composites with different HEA contents.

Samples	Density (g/cm^3^)	Hardness (HV)	YS (MPa)	UTS (MPa)
0 wt.% HEA	5.85 ± 0.03	451 ± 15	779 ± 45	2121 ± 55
5 wt.% HEA	5.96 ± 0.05	469 ± 21	1417 ± 52	2531 ± 51
10 wt.% HEA	6.06 ± 0.05	515 ± 25	1347 ± 54	2692 ± 45
15 wt.% HEA	6.12 ± 0.02	525 ± 21	1566 ± 42	2600 ± 48

## References

[1] Xu G.H., Zhang K.F., Huang Z.Q. (2012). The synthesis and characterization of ultrafine grain NiAl intermetallic. Adv. Powder Technol..

[2] Kaplin C., Ivanov R., Paliwal M., Jung I.H., Brochu M. (2014). The effect of nanostructure on the oxidation of NiAl. Intermetallics.

[3] Zhu H.X., Abbaschian R. (2000). Microstructures and properties of in-situ NiAl-Al_2_O_3_ functionally gradient composites. Compos. Part B Eng..

[4] Udhayabanu V., Ravi K.R., Murty B.S. (2011). Development of in situ NiAl-Al_2_O_3_ nanocomposite by reactive milling and spark plasma sintering. J. Alloys Compd..

[5] Arzt E., Grahle P. (1998). High temperature creep behavior of oxide dispersion strengthened NiAl intermetallics. Acta Mater..

[6] Yuan J., Zhang X., Li B., Wang X., Sun K. (2017). Microstructure and tribological behavior of NiAl/WC composites fabricated by thermal explosion reaction at 800 °C. J. Alloys Compd..

[7] Sheng L.Y., Yang F., Xi T.F., Guo J.T. (2013). Investigation on microstructure and wear behavior of the NiAl-TiC-Al_2_O_3_ composite fabricated by self-propagation high-temperature synthesis with extrusion. J. Alloys Compd..

[8] Ur S.C., Nash P. (2002). Secondary recrystallization and high temperature compressive properties of ODS MA NiAl. Scr. Mater..

[9] González-Carrasco J.L., Perez P., Adeva P., Chao J. (1999). Oxidation behaviour of an ODS NiAl-based intermetallic alloy. Intermetallics.

[10] Sheng L.Y., Yang F., Guo J.T., Xi T.F., Ye H.Q. (2013). Investigation on NiAl-TiC-Al_2_O_3_ composite prepared by self-propagation high temperature synthesis with hot extrusion. Compos. Part B Eng..

[11] Zhang Y., Zuo T.T., Tang Z., Gao M.C., Dahmen K.A., Liaw P.K., Lu Z.P. (2014). Microstructures and properties of high-entropy alloys. Prog. Mater. Sci..

[12] Karthik G.M., Panikar S., Ram G.J., Kottada R.S. (2017). Additive manufacturing of an aluminum matrix composite reinforced with nanocrystalline high-entropy alloy particles. Mater. Sci. Eng. A.

[13] Chen J., Niu P., Wei T., Hao L., Liu Y., Wang X., Peng Y. (2015). Fabrication and mechanical properties of AlCoNiCrFe high-entropy alloy particle reinforced Cu matrix composites. J. Alloys Compd..

[14] Zhang S., Wu C.L., Zhang C.H. (2015). Phase evolution characteristics of FeCoCrAlCuV_x_Ni high entropy alloy coatings by laser high-entropy alloying. Mater. Lett..

[15] Fu Z., Koc R. (2017). Processing and characterization of TiB_2_-TiNiFeCrCoAl high-entropy alloy composite. J. Am. Ceram. Soc..

[16] Wang L., Shen J., Shang Z., Fu H. (2014). Microstructure evolution and enhancement of fracture toughness of NiAl-Cr (Mo)-(Hf, Dy) alloy with a small addition of Fe during heat treatment. Scr. Mater..

[17] Liu E., Jia J., Bai Y., Wang W., Gao Y. (2014). Study on preparation and mechanical property of nanocrystalline NiAl intermetallic. Mater. Des..

[18] Liu Y., Wang J., Fang Q., Liu B., Wu Y., Chen S. (2016). Preparation of superfine-grained high entropy alloy by spark plasma sintering gas atomized powder. Intermetallics.

[19] Tsai K.Y., Tsai M.H., Yeh J.W. (2013). Sluggish diffusion in Co-Cr-Fe-Mn-Ni high-entropy alloys. Acta Mater..

[20] Kucza W., Dąbrowa J., Cieślak G., Berent K., Kulik T., Danielewski M. (2018). Studies of “sluggish diffusion” effect in Co-Cr-Fe-Mn-Ni, Co-Cr-Fe-Ni and Co-Fe-Mn-Ni high entropy alloys; determination of tracer diffusivities by combinatorial approach. J. Alloys Compd..

[21] Rezaei M.R., Razavi S.H., Shabestari S.G. (2016). Development of a novel Al-Cu-Ti metallic glass reinforced Al matrix composite consolidated through equal channel angular pressing (ECAP). J. Alloys Compd..

[22] Grosdidier T., Ji G., Launois S. (2007). Processing dense hetero-nanostructured metallic materials by spark plasma sintering. Scr. Mater..

[23] Liu X., Yin H., Xu Y. (2017). Microstructure, Mechanical and Tribological Properties of Oxide Dispersion Strengthened High-Entropy Alloys. Materials.

[24] Povarova K.B., Kazanskaya N.K., Drozdov A.A., Morozov A.E. (2006). Physicochemical laws of the interaction of nickel aluminides with alloying elements: I. Formation of nickel aluminide-based solid solutions. Russ. Metall. (Met.).

[25] Chérif A., Bachaga T., Saurina J., Suñol J.J., Khitouni M., Makhlouf T. (2016). Morphology and structure effect of Ti additive on the solid-state reaction between Ni and Al powders during mechanical alloying. Int. J. Adv. Manuf. Technol..

[26] Anderson I.M., Duncan A.J., Bentley J. (1999). Site-distributions of Fe alloying additions to B2-ordered NiAl. Intermetallics.

[27] Zhang Q., Xiao B.L., Liu Z.Y., Ma Z.Y. (2011). Microstructure evolution and elemental diffusion of SiCp/Al-Cu-Mg composites prepared from elemental powder during hot pressing. J. Mater. Sci..

[28] Huang L., Geng L., Li A.B., Yang F.Y., Peng H.X. (2009). In situ TiBw/Ti-6Al-4V composites with novel reinforcement architecture fabricated by reaction hot pressing. Scr. Mater..

[29] Liu E., Gao Y., Jia J., Bai Y., Wang W. (2014). Microstructure and mechanical properties of in situ NiAl-Mo_2_C nanocomposites prepared by hot-pressing sintering. Mater. Sci. Eng. A.

[30] Li S., Qi L., Zhang T., Zhou J., Li H. (2016). Interfacial microstructure and tensile properties of carbon fiber reinforced Mg-Al-RE matrix composites. J. Alloys Compd..

[31] Yin H., Xu Y., Li X., Chang W., Zhou Y. (2017). Design of friction and wear resistant titanium-and cobalt-modified nickel-base repair alloys by spray forming. Mater. Des..

[32] Jaeger R.M., Kuhlenbeck H., Freund H.J., Wuttig M., Hoffmann W., Franchy R., Ibach H. (1991). Formation of a well-ordered aluminium oxide overlayer by oxidation of NiAl (110). Surf. Sci..

[33] Scudino S., Liu G., Prashanth K.G., Bartusch B., Surreddi K.B., Murty B.S., Eckert J. (2009). Mechanical properties of Al-based metal matrix composites reinforced with Zr-based glassy particles produced by powder metallurgy. Acta Mater..

